# Feature Fusion Based on Graph Convolution Network for Modulation Classification in Underwater Communication

**DOI:** 10.3390/e25071096

**Published:** 2023-07-21

**Authors:** Xiaohui Yao, Honghui Yang, Meiping Sheng

**Affiliations:** School of Marine Science and Technology, Northwestern Polytechnical University, Xi’an 710072, China

**Keywords:** automatic modulation classification, underwater acoustic communication signals, graph convolution network, feature fusion

## Abstract

Automatic modulation classification (AMC) of underwater acoustic communication signals is of great significance in national defense and marine military. Accurate modulation classification methods can make great contributions to accurately grasping the parameters and characteristics of enemy communication systems. While a poor underwater acoustic channel makes it difficult to classify the modulation types correctly. Feature extraction and deep learning methods have proven to be effective methods for the modulation classification of underwater acoustic communication signals, but their performance is still limited by the complex underwater communication environment. Graph convolution networks (GCN) can learn the graph structured information of the data, making it an effective method for processing structured data. To improve the stability and robustness of AMC in underwater channels, we combined the feature extraction and deep learning methods by fusing the multi-domain features and deep features using GCN. The proposed method takes the relationships among the different multi-domain features and deep features into account. Firstly, a feature graph was built using the properties of the features. Secondly, multi-domain features were extracted from the received signals and deep features were extracted from the signals using a deep neural network. Thirdly, we constructed the input of GCN using these features and the graph. Then, the multi-domain features and deep features were fused by the GCN. Finally, we classified the modulation types using the output of GCN by way of a softmax layer. We conducted the experiments on a simulated dataset and a real-world dataset, respectively. The results show that the AMC based on GCN can achieve a significant improvement in performance compared to the current state-of-the-art methods. Our approach is robust in underwater acoustic channels.

## 1. Introduction

AMC has been an important method with which to identify the modulation types of the received signals in underwater communication scenarios; this is useful for the monitoring and identification of communication interference, which are core technologies in spectrum surveillance and underwater acoustic countermeasures. The advanced AMC technology has a broad application prospect in the underwater unmanned platform [1]. However, the complexity of underwater communication means the underwater acoustic channel is full of multi-path fading and ocean ambient noise, which can decrease the AMC performance of underwater acoustic communication signals significantly.

The AMC methods include two categories: the maximum likelihood ratio algorithm and the feature extraction algorithm. Due to the high computational complexity of the maximum likelihood ratio algorithm, most of the current studies on AMC focus on the feature extraction algorithm. The commonly used feature extraction methods in AMC include instantaneous statistics features (envelop, frequency, phase, etc.) [2], high-order cumulant features (HOC) [3,4], spectrum features [5,6,7], cyclostationary statistics features (CS) [8,9,10], and wavelet features [4,11], etc. In recent years, some new feature extraction methods based on entropy have shown effectiveness in underwater signals processing [12,13]. These feature extraction methods are always followed by a classifier; the common classifiers include neural network classifiers [14], support vector machine (SVM) [15], decision tree classifiers [16], and so on. Some applications of these feature extraction methods and classifiers have been used in AMC approaches for underwater acoustic communication signals. Zhao [17] introduced the Stockwell-transform and SVM into modulation classification in underwater acoustic channels; Stockwell-transform is a kind of spectrum feature. Sanderson [8] proposed hierarchical blind modulation classification for underwater acoustic communication signals; they used second order cyclostationary features to classify the binary phase shift keying (BPSK) and non-BPSK signals. Wu [9] proposed a modulation detection scheme for underwater acoustic communication signals through cyclostationary analysis; they extracted cyclic frequency/frequency-peak ratio to identify the modulation types. Ge [18] used HOC features and a spectrum correlation function for AMC of underwater acoustic communication signals. The performance of the AMC based on the feature extraction algorithm depends on the quality of the features.

As deep learning has shown remarkable results in many fields, many deep learning neural networks (DNN) have been proposed for various tasks. Convolution neural networks (CNN) [19] are used to process computer vision and natural language, and to build some advanced deep learning models, such as ResNet [20], GoogleNet [21], VGGNet [22], and generative adversarial networks (GAN) [23], etc. Recurrent neural networks (RNN) [24] are always used to process time series data; widely used variants of the RNN include long short-term memory (LSTM) [25] and gate recurrent unit (GRU) [26]. Some AMC methods based on deep learning theory have been proposed in recent years. DNNs can learn high-level features from raw data automatically without much prior knowledge, or can also accept the features from a feature extraction algorithm and work as a classifier. Yao [1] proposed an AMC method based on deep complex networks. They built two complex physical signal processing layers to improve the performance of AMC in underwater acoustic communication. Zhang [27] proposed an AMC method based on a multi-scale network to address the inter-class diversity problem. Zhou [28] proposed an AMC relation network for AMC under few-shot conditions. Yao [29] used GAN to enhance the signals and showed good robustness under different underwater acoustic channels. O’Shea [30] carried out research on the performance of deep learning; the effects of carrier frequency offset, symbol rate, and multi-path fading were considered. F. Wang [31] combined deep learning and a zero-center normalized instantaneous amplitude tightness characteristic parameter to overcome the intra-class diversity problem; the proposed method improved the classification performance of quadrature amplitude modulation signals. Yu [32] used LSTM for AMC of non-cooperative underwater acoustic communication signals. Jiang [33] used a sparse autoencoder network to realize data transfer for the AMC of underwater acoustic communication signals. Ding [34] proposed a deep neural network for the AMC of underwater acoustic communication signals that combined the CNN with LSTM; they used CNN to learn the time domain IQ data and LSTM to learn the amplitude and phase data.

In recent years, the underlying relationships among data have attracted more and more attention in several areas of machine learning. There have been studies that attempted to exploit the graph structure information in data processing [35]. A graph convolution network (GCN) builds a neural network based on the topology of the data graph. GCN can be used to classify elements of the graph or the graph itself. There have been many applications of GCN in the field of identification. Long [36] proposed a multi-modal relational graph network to dynamically integrate visual and kinematics information to boost gesture recognition accuracy in robotic surgery. Kipf [37] presented a graph convolution network for the semi-supervised classification of graph-structured data; the performance of the proposed model was validated on different datasets. In the field of AMC, Xuan [38] proposed an adaptive visibility graph algorithm to map a time series into a graph adaptively; they used the proposed method and GCN to achieve modulation classification of radio signals.

In this paper, we proposed a new method for AMC of underwater acoustic communication signals using GCN. In the past few years, traditional feature extraction methods have been proven effective in some conditions. To improve the stability and robustness of AMC in underwater scenarios, we used GCN to integrate the multi-domain features and deep features of the received underwater acoustic communication signals. The multi-domain features come from HOC, CS, and high order moment (HOM). We extracted multi-domain features of the received signals and learned the deep features from the signals. A feature graph was built using the properties of the features. Then, the multi-domain features and deep features were fused by the GCN. Finally, we classified the modulation type using the fused features. Our contributions are as follows:

1. We adopted GCN to AMC to improve the stability and robustness of AMC in underwater communication scenarios. GCN was used to fuse the multi-domain features and deep features of the received signals.
2. To take the relationships between multi-domain features and deep features into account, we built a graph of the multi-domain features and deep features using their properties.
3. The performance of the proposed method was validated using the simulated dataset in different underwater acoustic channels and a real-world dataset.

This paper is organized as follows. Section 2 introduces the proposed AMC method of underwater acoustic communication signals based on GCN. In Section 3, we evaluated the performance of the proposed method with a series of contrastive experiments using simulation and real-world datasets. Finally, the conclusion of the paper is given in Section 4.

## 2. Materials and Methods

### 2.1. Multi-Domain Features

We chose three kinds of features extraction methods to extract the multi-domain features from the received signals. These feature extraction methods included HOC, CS, and HOM.

#### 2.1.1. High-Order Cumulant

High-order cumulant (HOC) [3,4,39] is a common feature extraction method for AMC. Since the cumulants of an order higher than 3 for a Gaussian distribution are zero, the HOC of a signal with additive white Gaussian noise is ideally the HOC of the signal without noise. Given a received signal x(t), the *p*-th order mixing moment can be expressed as:

(1) $$M_{pq}=E\left[x{\left(t\right)}^{p-q}x^{*}{\left(x\right)}^{q}\right],$$

where E[•] is the expected value operator, ∗ is the complex conjugate. The different order HOC features used in our work can be expressed as:

(2) $$C_{20}=M_{20}$$

(3) $$C_{21}=M_{21}$$

(4) $$\;C_{40}=M_{40}-3M_{20}^{2}$$

(5) $$\;C_{41}=M_{41}-3M_{21}M_{20}$$

(6) $$\;C_{42}=M_{42}-M_{20}^{2}-2M_{21}^{2}$$

(7) $$\;C_{60}=M_{60}-15M_{20}M_{40}+30M_{20}^{2}$$

(8) $$\;C_{61}=M_{61}-5M_{40}M_{21}-10M_{20}M_{41}+30M_{21}M_{20}^{2}$$

(9) $$\;C_{63}=M_{63}-9M_{42}M_{21}-9M_{20}^{2}M_{21}+12M_{21}^{3}$$

(10) $$\;C_{80}=M_{80}-28M_{20}M_{60}-35M_{40}^{2}+420M_{20}^{2}M_{40}-630M_{20}^{4}.$$

The relationships among these HOC features were used to construct the graph of the features. It is obvious that each feature has a relationship with x(t). The internal relationships can be obtained according to Equations (2)–(10) and can be expressed in Table 1.

#### 2.1.2. Cyclostationary Statistics

Cyclostationary statistics (CS) is an important tool for performing signal detection, modulation classification, signal parameter estimation, etc. CS is based on the fact that communications signals are not accurately described as stationary, but rather more appropriately modeled as cyclostationary. We used second-order CS features in the proposed framework, including spectral correlation density (SCD), which can be denoted as $S_{X}^{\alpha }\left(f\right)$ [10,40]. $S_{X}^{\alpha }\left(f\right)$ of a signal x(t) is defined as:

(11) $$\;\begin{array}{cc} \; & S_{X}^{\alpha }\left(f\right)=\lim_{T\to \infty }\lim_{\Delta T\to \infty }\frac{1}{\Delta T}\int_{-\frac{1}{\Delta T}}^{\frac{1}{\Delta T}}\frac{1}{T}X_{T}\left(t,f+\frac{\alpha }{2}\right)X_{T}^{*}(t,f-\frac{\alpha }{2})\;dt \end{array}$$

(12) $$\begin{array}{cc} \; & X_{T}\left(t,f\right)=\int_{t-\frac{T}{2}}^{t+\frac{T}{2}}x\left(u\right)e^{j2\pi fu}\;du, \end{array}$$

where α is the cyclic frequency. The normalized version of the SCD is spectral coherence function (SCF), which can be calculated by:

(13) $$C_{X}^{\alpha }\left(f\right)=\frac{S_{X}^{\alpha }\left(f\right)}{{\left[S_{X}^{0}\left(f+\frac{\alpha }{2}\right)*S_{X}^{0}\left(f-\frac{\alpha }{2}\right)\right]}^{\frac{1}{2}}}.$$

It is obvious that $S_{X}^{\alpha }\left(f\right)$ and $C_{X}^{\alpha }\left(f\right)$ of a signal can be visualized as images. To simplify the CS features, we used the frequency profile as well as the cycle frequency profile from $C_{X}^{\alpha }\left(f\right)$ [10]:

(14) $$\begin{array}{cc} \; & I\left(\alpha \right)=\max_{f}\left|C_{X}^{\alpha }\left(f\right)\right| \end{array}$$

(15) $$\begin{array}{cc} \; & I\left(f\right)=\max_{\alpha }\left|C_{X}^{\alpha }\left(f\right)\right|. \end{array}$$

#### 2.1.3. High Order Moment

High order moment (HOM) [41] is a kind of spectrum feature. HOM is associated with the modulation order and it is often used for intra-class classification of phase shift keying modulation signals. The *K* order HOM ($U^{K}\left(f\right)$) of a signal x(t) can be represented as:

(16) $$U^{K}\left(f\right)=F\left(x^{K}\left(t\right)\right),$$

where $F\left(•\right)$ denotes the Fourier transform function and *K* is the order of HOM. When *K* is an integral multiple of the modulation order, there will be distinct lines in $U^{K}\left(f\right)$. $U^{2}\left(f\right)$ and $U^{4}\left(f\right)$ will be used in the following work.

### 2.2. The Proposed AMC Method

**Figure 1:**
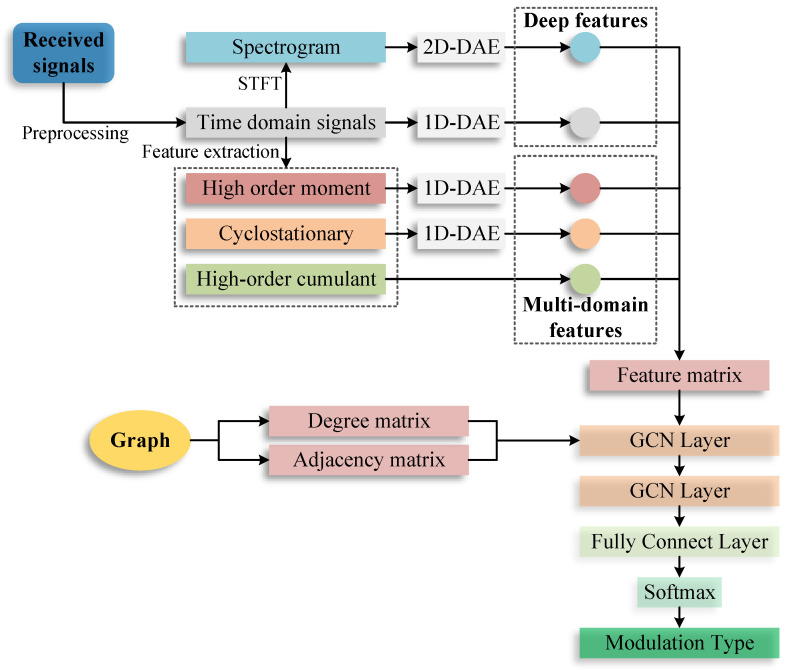
Framework of the proposed AMC method based on GCN.

The framework of the proposed method is illustrated in Figure 1. The graph was built based on the properties of the multi-domain features and deep features. The multi-domain features were extracted using different feature extraction methods. Different deep features were learned from the time domain and short-time Fourier transform (STFT) of the received signals, respectively. These features and the graph were used to construct the input matrices of GCN. We used GCN to fuse these features and used a softmax layer to classify the modulation types.

#### 2.2.1. Graph Convolution Network

A graph convolution network (GCN) was used to learn features from a graph. Unlike CNNs, which operate on a local region in an image, in GCN, the convolutional operations compute the response at a node based on the neighboring nodes defined by the adjacency graph. A graph can be denoted as G=(V,E), where V is the set of nodes and E is the set of edges. Nodes in a graph represent objects or concepts, and edges represent their relationships. The adjacency matrix is denoted as A, the node feature matrix is $F\in R^{n\times d}$, *n* is the number of the nodes, and *d* is the length of the node feature. The propagation rule in GCN can be expressed as:

(17) $$F^{\left(l+1\right)}=\sigma \left({\tilde{D}}^{-\frac{1}{2}}\tilde{A}{\tilde{D}}^{-\frac{1}{2}}F^{\left(l\right)}W^{\left(l\right)}\right),$$

where $\tilde{A}=A+I_{N}$ is the adjacency matrix of the graph G with added self-connections. $I_{N}$ is the identity matrix, $\tilde{D}$ is the degree matrix, $W^{\left(l\right)}$ is a layer-specific trainable weight matrix, $F^{\left(l\right)}$ is the matrix of activations in the *l*-th layer, and $\sigma \left(•\right)$ denotes an activation function; we used a linear rectification unit (ReLU) as the activation function.

#### 2.2.2. Features Fusion Based on GCN

(a) Build graph for the features.

**Figure 2:**
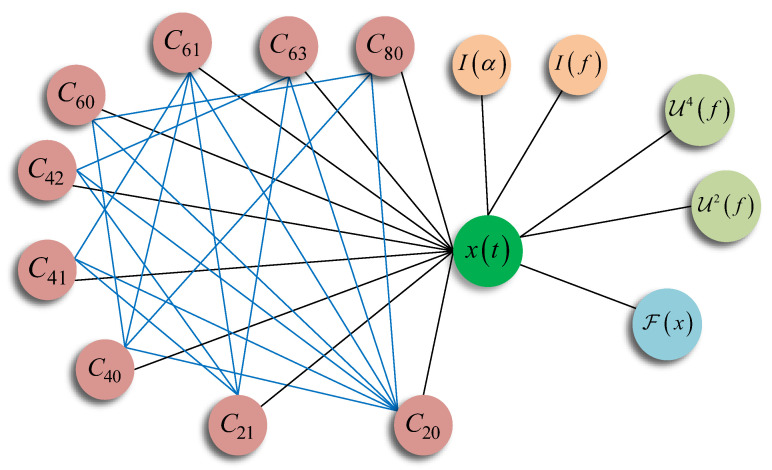
The graph of the multi-domain features and deep features. The graph is undirected, the black edges denote the relationships between different domain, the blue edges denote the relationships between the nodes belonging to the same domain.

We built an undirected graph of the features. There are 15 nodes in the graph (N=15), which include time domain signal x(t), STFT F(x), nine HOC features, two CS features and two HOM features. We denote each node as $v_{i}$ and the node-feature pairs are shown in Table 2. The graph was built using the properties of the features. The nodes were connected based on the mathematics of the feature extraction algorithms, for example, $C_{80}$ was calculated using x(t), $C_{20}$, $C_{40}$ and $C_{60}$, and there were four edges between $C_{80}$ and the other four nodes. The graph of the these features is shown in Figure 2.

(b) Extract features for each node.

**Figure 3:**
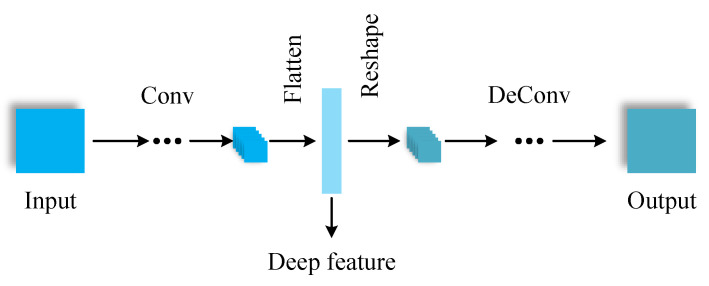
The architecture of the DAE.

Deep features include features from the time domain and STFT of the received signals. We used deep autoencoder networks (DAE) [42] to extract the deep features from the time domain signals and their STFT. The architecture of DAE is shown in Figure 3. Since the time domain signal is a 1D complex vector and the STFT is a 2D matrix, we used 1D-DAE and 2D-DAE to extract deep features from the time domain and STFT, respectively. The real part and the imaginary part of the time domain signal were treated as two channels. The deep features of these two DAE are 1D vectors and the length is 128.

The multi-domain features were extracted using the corresponding feature extraction methods. Each HOC feature has only one value. The CS features and HOM features are all 1D vectors. We used 1D-DAE to compress these features to have same length as the deep features.

(c) Construct the input of GCN.

The input of GCN includes three matrices: adjacency matrix $\tilde{A}$, degree matrix $\tilde{D}$, and feature matrix degree matrix F. $\tilde{A}$ and $\tilde{D}$ can be extracted from the feature graph. The number of the nodes is 15 and they were sorted in the order shown in Table 2. $\tilde{A}$ is used to express the relationships between the nodes; element $\left(v_{i},v_{j}\right)$ represents the relationship between node *i* and node *j*; $(v_{i},v_{j})=1$ indicates that the two nodes are related; $(v_{i},v_{j})=0$ indicates that the two nodes are not related when i=j, $(v_{i},v_{j})=1$. Then, $\tilde{A}$ can be repressed as Equation (18). The rows and columns correspond to the nodes in Table 2; they are separated by dotted lines according to the corresponding feature domains.

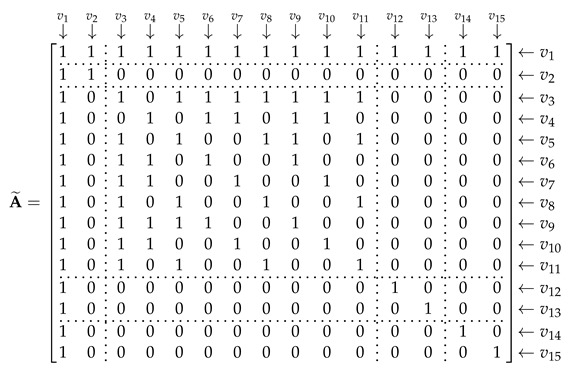
(18)


$\tilde{D}$ is a diagonal matrix, which can be expressed as:

(19) $$\tilde{D}=\mathrm{diag}\left(15,2,9,6,6,5,5,5,6,5,5,2,2,2,2\right).$$

The size of F was set to 15 × 128. To build the feature matrix, the length of each feature should be 128. For the HOC features, we used the zero-padding to supplement their length to 128.

(d) Feature fusion and modulation classification.

We used two GCN layers to learn features from the input graph and features. The ${\tilde{D}}^{-\frac{1}{2}}\tilde{A}{\tilde{D}}^{-\frac{1}{2}}$ in Equation (17) can be calculated in a pre-processing step. The output of the last GCN layer was flatted to a 1D vector. Then, we used a softmax layer to classify the modulation types. A fully connection layer was used to connect the GCN layer and the softmax layer. The weights of these layers were trained using gradient descent.

## 3. Experiments and Discussion

We conducted a series of contrastive experiments in this section to verify the performance of the proposed AMC method:

(1) We analyzed the influence of the different features.
(2) We analyzed the influence of the edges inside HOC.
(3) We compared the performance of the proposed method with other AMC methods.
(4) The performance of the proposed method was verified using real-world underwater acoustic communication signals.

The results in this section were the average values over multiple runs.

### 3.1. Dataset and Parameters

#### Signals Generation

We considered several commonly used modulation types in underwater acoustic communication scenarios, including frequency shift keying (FSK) (2FSK, 4FSK, 8FSK), phase shift keying (PSK) (BPSK, QPSK, 8PSK), and quadrature amplitude modulation (QAM) (16QAM, 32QAM, 64QAM). In the simulation condition, the SNR ranges from −9 dB to 21 dB with an interval of 3 dB. The received signals were expressed as the sampled complex baseband, the dimension of each sample was 3000 × 2, and the duration was 0.25 s. The number of each modulation type at each SNR was 10,000, then the total number of samples was 990,000. Of the samples, 75% were used as training signals and 25% were used as testing samples. The parameters of each modulation type are shown in Table 3, the frequency separation of FSK modulation was 200 Hz.

**Figure 4:**
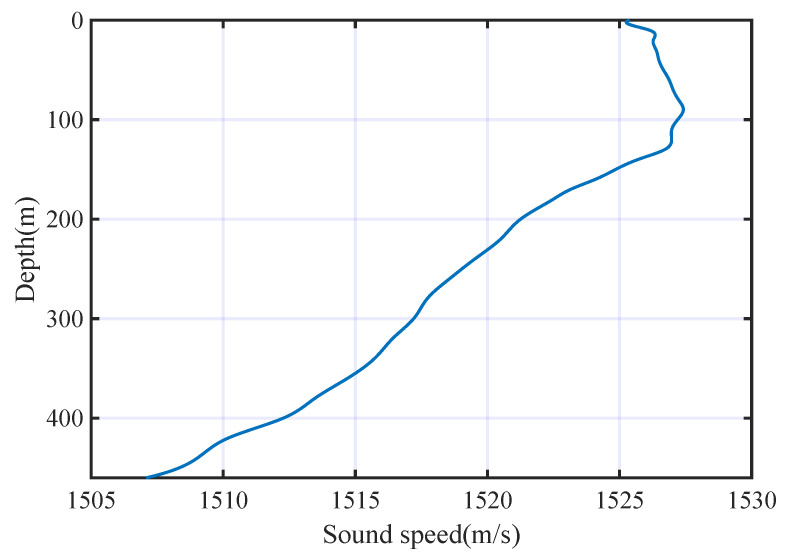
Sound velocity profile.

We used the simulated underwater acoustic channels with multi-path fading. The sound velocity profile is shown in Figure 4. The depth of the sea is 460 m.

**Figure 5:**
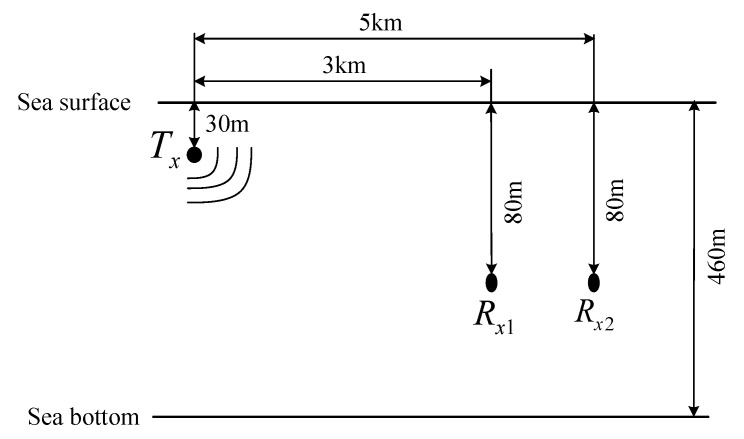
Diagram of underwater acoustic channel.

There was one transmitter ($T_{x}$) and two receivers ($R_{x1}$ and $R_{x2}$) in the simulated underwater acoustic communication channel, as shown in Figure 5. The horizontal distances between the transmitter and the two receivers were 3 km and 5 km, respectively. The depths of the transmitter and receivers were 30 m and 80 m, respectively.

**Figure 6:**
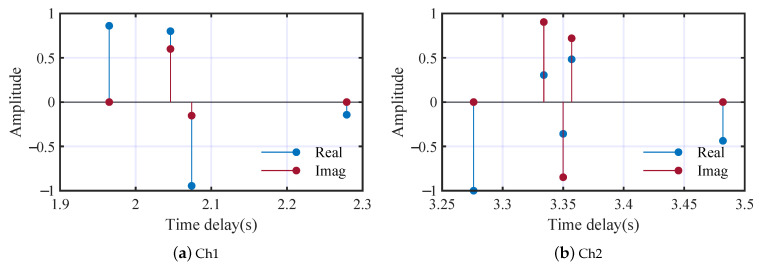
Time delays and amplitudes of the two multi-path fading channels.

The time delays and amplitudes of the two multi-path fading channels are shown in Figure 6, in which the modules of the amplitudes are normalized to [0,1].

### 3.2. Experiment Results Analysis

A series of contrastive experiments was carried out in the following work. In each simulation experiment, we calculated the classification accuracy at each SNR point and the average accuracy at all SNR, which can be expressed as:

(20) $$\bar{Acc}=\frac{1}{N_{snr}}\sum_{i=1}^{N_{snr}}Acc_{i}.$$

$Acc_{i}$ is the classification accuracy at the *i*-th SNR point from −9 dB to 21 dB, $\bar{Acc}$ is the average accuracy at all SNR, and $N_{snr}$ is the number of SNR points. We analyzed the performance in the contrastive experiments mainly using the average accuracy.

#### 3.2.1. The Analysis of the Influence of the Different Features

We used an ablation experiment to analyze the influence of the different features and verify the effectiveness of the proposed method. The features were extracted individually from the signals. In the following contrastive experiments, the features coming from different domains were replaced by white Gaussian noise (WGN) in turn. Each experiment was carried out in the two multi-path channels, respectively.

(a) Baseline performance.

**Figure 7:**
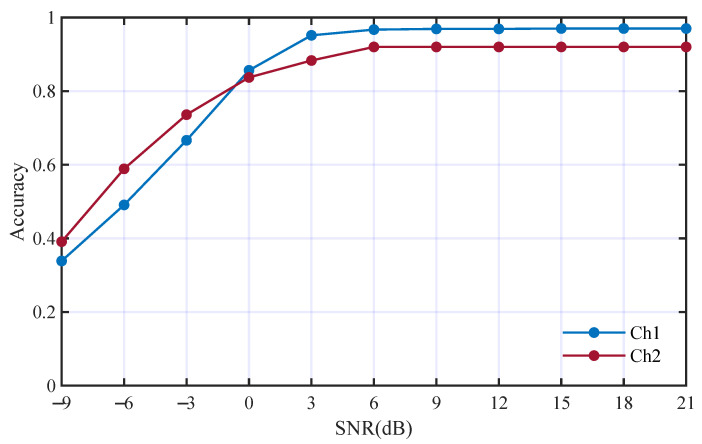
Performance of proposed method in the two underwater multi-path channels.

The performance of the proposed method with all features was used as a baseline and the classification is shown in Figure 7.

**Figure 8:**
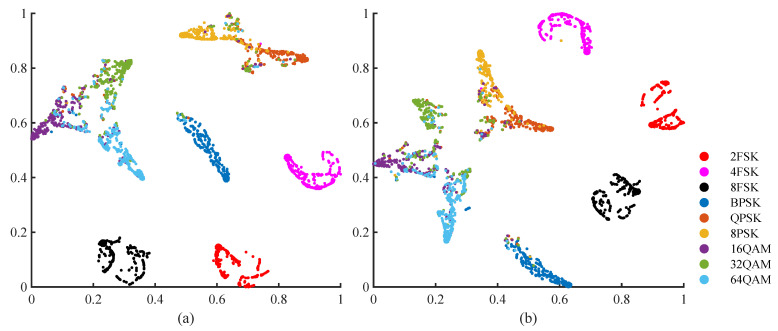
Visualization of the features of the fully connected layer: (**a**) features are learned from the signals in Ch1 (SNR = 6 dB); (**b**) features are learned from the signals in Ch2 (SNR = 6 dB).

The mean accuracies in Ch1 and Ch2 are 82.9% and 81.4%, respectively. To analyze the classification of each modulation type, we visualized the features from the fully connected layer using t-SNE [43], as shown in Figure 8. We can see from Figure 8 that, in the multi-path fading channels, the classification errors mainly occur among different modulation orders of the same modulation mode.

(b) Deep feature of time domain.

**Figure 9:**
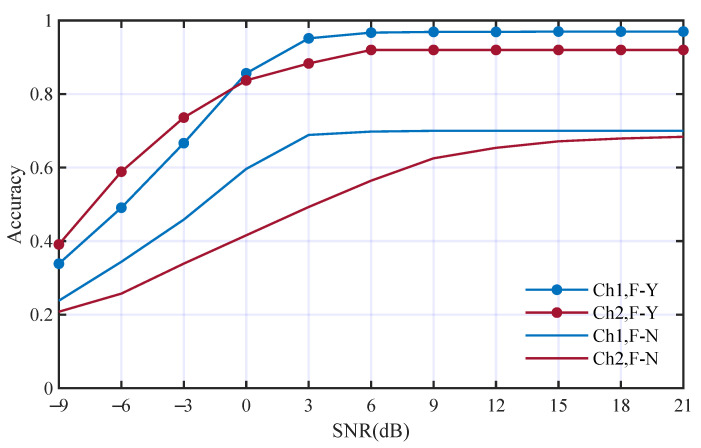
Performance comparison with and without deep features from the time domain; F-Y and F-N mean with and without such deep features, respectively.

To analyze the contribution of deep feature from time domain, we first replaced the deep features from the time domain with WGN and other conditions were kept the same. The performance comparison is shown in Figure 9. The average accuracies using deep features from the time domain in Ch1 and Ch2 are 82.9% and 81.4%. The average accuracies without using deep features from the time domain in Ch1 and Ch2 are 59.3% and 50.8%. The accuracies without using deep features from the time domain decrease to 23.6% and 30.6% in the two channels, respectively. It is obvious that the deep features from the time domain make great contributions to the AMC performance.

(c) Deep features of STFT.

**Figure 10:**
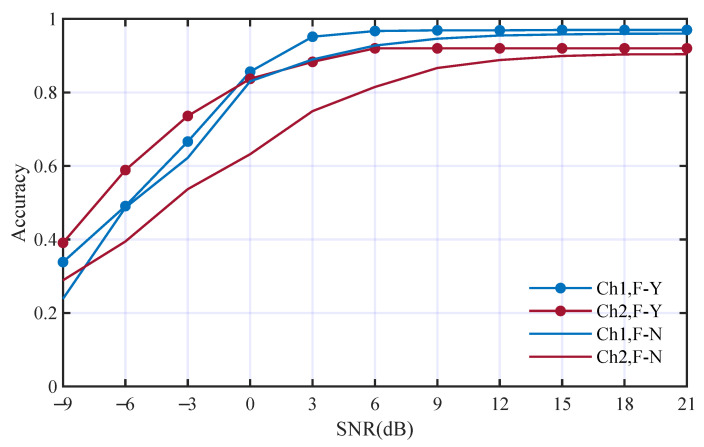
Performance comparison with and without deep features from STFT; F-Y and F-N mean with and without such deep features, respectively.

Secondly, the deep features from STFT were replaced by WGN and other conditions were kept the same. Figure 10 has shown the performance comparison. The average accuracies without using deep features from STFT in Ch1 and Ch2 are 79.7% and 71.6%. The accuracies without using deep features from STFT decrease to 3.2% and 9.8% in the two channels, respectively. The influence of the deep features from STFT was smaller than that of the time domain.

(d) HOC features.

**Figure 11:**
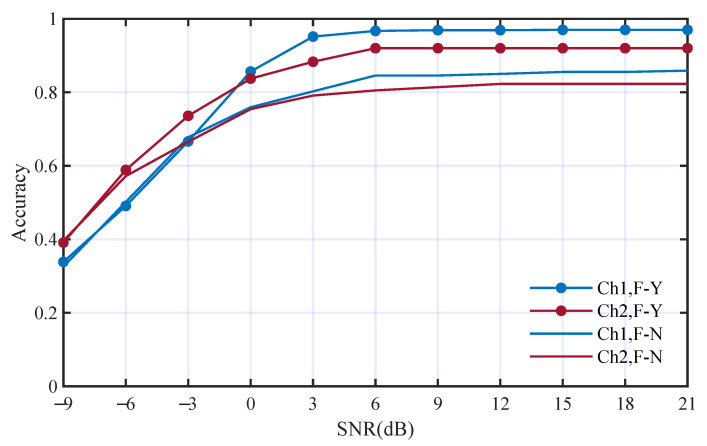
Performance comparison with and without HOC features; F-Y and F-N mean with and without HOC features, respectively.

Thirdly, the nine HOC features were replaced by WGN and other conditions were kept the same. The performance comparison is illustrated in Figure 11. The average accuracies without using HOC features in Ch1 and Ch2 are 74.3% and 73.5%. The accuracies without using HOC features decrease to 8.6% and 7.9% in the two channels, respectively. Figure 11 shows that the HOC features mainly influence the AMC performance at a higher SNR.

(e) CS features.

**Figure 12:**
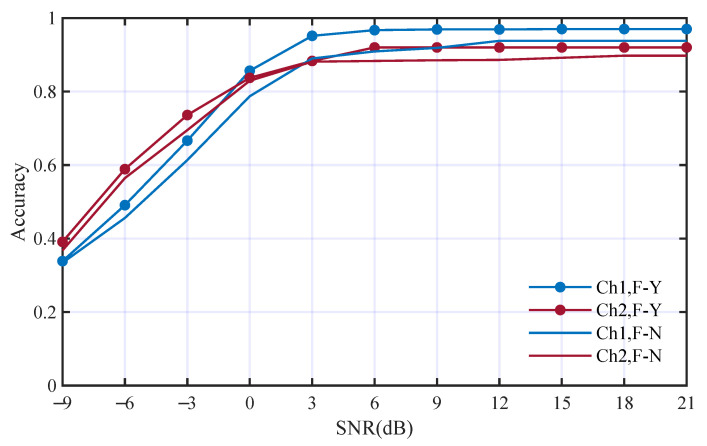
Performance comparison with and without CS features; F-Y and F-N mean with and without CS features, respectively.

Fourthly, the two CS features were replaced by WGN and other conditions were kept the same. The performance comparison is illustrated in Figure 12. The average accuracies without using HOC features in Ch1 and Ch2 are 78.7% and 78.9%. The accuracies without using CS features decrease to 4.2% and 2.5% in the two channels, respectively.

(f) HOM features.

**Figure 13:**
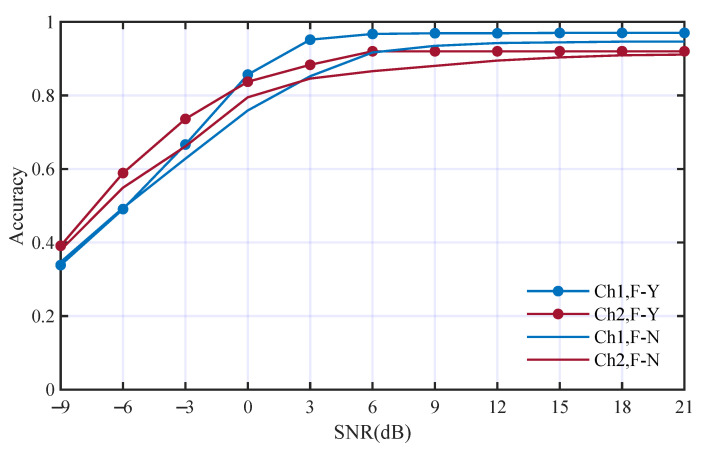
Performance comparison with and without HOM features; F-Y and F-N mean with and without HOM features, respectively.

Finally, the two HOM features were replaced by WGN and other conditions were kept the same. The performance comparison is shown in Figure 13. The average accuracies without using CS features in Ch1 and Ch2 are 79.2% and 78.1%. The accuracies without using CS features decrease to 3.7% and 3.3% in the two channels, respectively.

The summary of this ablation experiment is shown in Table 4. Table 4 shows that the multi-domain feature fusion based on GCN is quite effective for the AMC of underwater acoustic communication signals. All the features make contributions to the AMC performance. The deep features from the time domain are the most indispensable for an exact classification.

#### 3.2.2. The Analysis of the Influence of the Edges Inside HOC

Nine features were extracted using the HOC algorithm. The relationships among these features are complex; we constructed these edges based on the calculation relationships of such features. To analyze the influence of these edges, a contrastive experiment was carried out. In this experiment, a new adjacency matrix ${\tilde{A}}_{1}$ and degree matrix ${\tilde{D}}_{1}$ were used as the input of GCN. Since we would not consider the edges inside HOC, ${\tilde{A}}_{1}$ and ${\tilde{D}}_{1}$ can be expressed as:

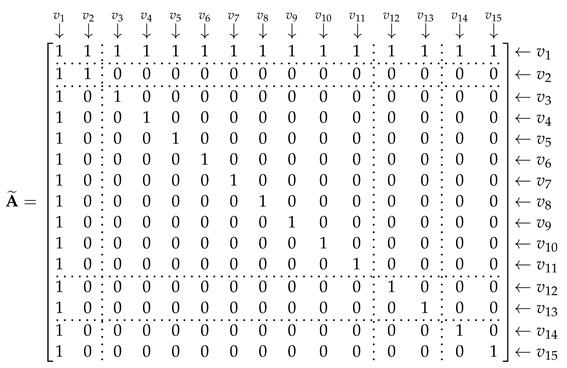
(21)

(22) $$\tilde{D}=\mathrm{diag}\left(15,2,2,2,2,2,2,2,2,2,2,2,2,2,2\right).$$

**Figure 14:**
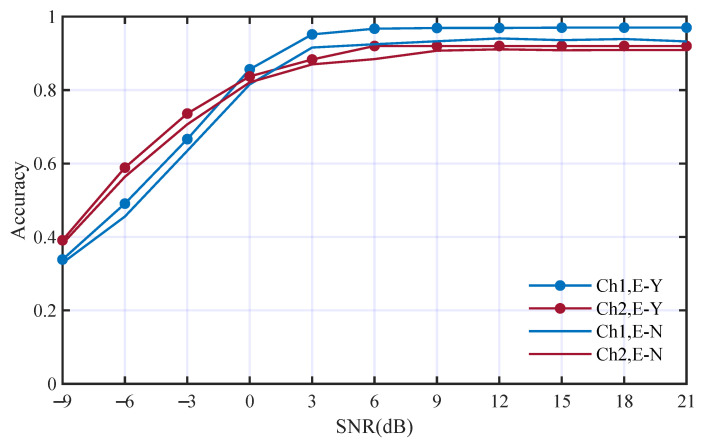
Performance comparison with and without edges inside HOM features; E-Y and E-N mean with and without such edges respectively.

The network was trained in the same way as the baseline. The classification results are shown in Figure 14, in which the baseline performance was used for comparison.

The average accuracies without using the edge inside HOC are 79.5% and 78.4%. The comparison of the accuracies is shown in Table 5. The accuracies without using edges inside HOC decrease to 3.4% and 3.0% in the two channels, respectively. The comparison result shows that making use of the relationships between HOC features can improve the classification performance.

#### 3.2.3. Comparison with Other State-of-the-Art AMC Methods

**Figure 15:**
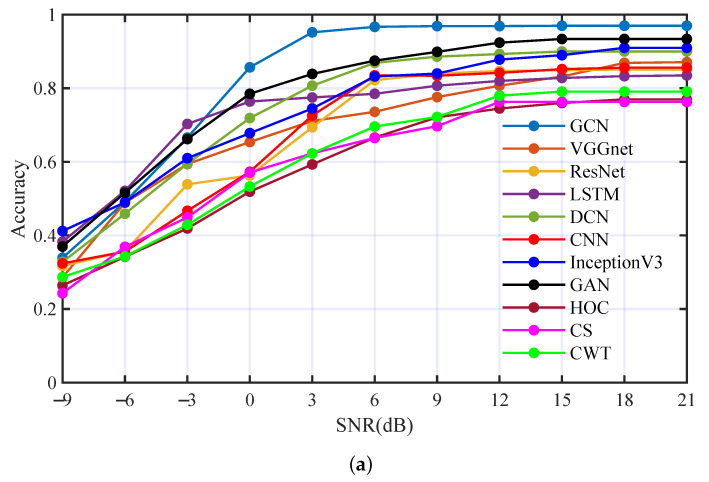

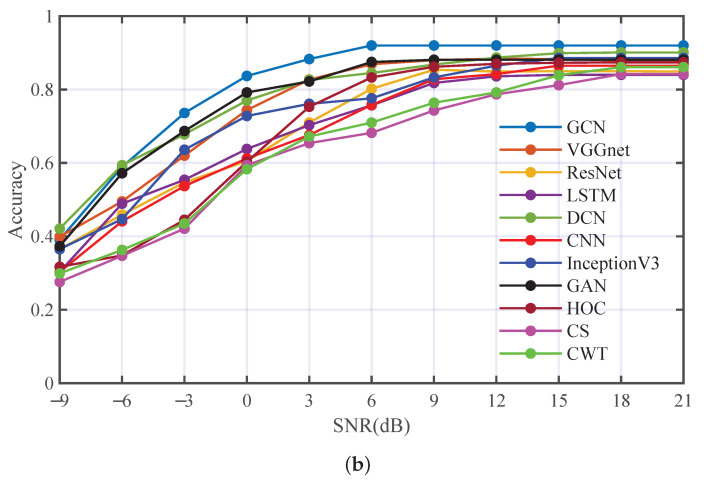
Performance comparison with state-of-the-art AMC methods: (**a**) comparison result in Ch1, (**b**) comparison result in Ch2.

To demonstrate the effectiveness of the proposed AMC method based on GCN, we compared the performance of the proposed method with those state-of-the-art AMC methods. The achieved methods include deep learning methods (basic CNN [44], InceptionV3 [45], GAN [29], VGGnet [30], ResNet [46,47], LSTM [48,49], deep complex network (DCN) [1]), and feature extraction methods (HOC [3,4] using an SVM classifier, CS [50] with a neural network classifier, and continuous wavelet transform (CWT) [11,51] with an SVM classifier). We carried out the comparison experiments in Ch1 and Ch2, respectively. The performance comparison is shown in Figure 15 and the average accuracy comparison is shown in Table 6. The proposed method has obvious advantages in both underwater acoustic channels.

#### 3.2.4. Performance Analysis Using Real-World Dataset

To verify the performance of the proposed AMC method in a real-world underwater scenario, we carried out an experiment using the real-world underwater acoustic communication dataset. This dataset was recorded in the South China Sea. The data were recorded using an omnidirectional hydrophone placed about 10 m under the surface, the transmitter was about 3 km away from the receiver, and the relative speed of the transmitter and receiver was less than 5 m/s. The modulation types of this dataset were 2FSK, 4FSK, BPSK, QPSK, 16QAM, and 32QAM. The SNR of the received signals was about 3–5 dB. The number of each modulation type was 100. The classification results are shown in Table 7. The proposed method can classify the real-world dataset well; the average accuracy of this dataset is 75.3%.

#### 3.2.5. Computational Cost Analysis

**Figure 16:**
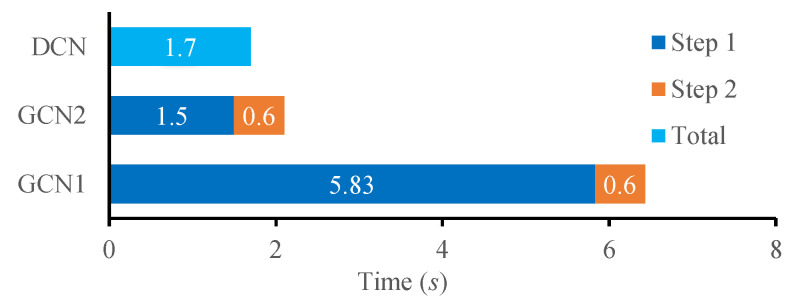
Computational cost comparison of different methods. GCN1 denotes the process of the first step without GPU acceleration, GCN2 denotes the process of the first step with GPU acceleration.

Computational cost is an important performance metric for AMC. To analyze the computational cost of our proposed AMC method, we calculated the time consumed by the modulation types prediction process. The prediction process of the proposed method includes two steps. The first step is to extract the multi-domain features and the deep features and the second step is the forward propagation of the GCN and its subsequent network layers. The first step typically involves complex calculations and requires a significant amount of computation. The second step was implemented in the CUDA environment, which consumed fewer computing resources with GPU acceleration. In order to accelerate the computational speed, we redesigned the calculation operation of feature extraction using TensorFlow in the CUDA environment. Thus, we could not only accelerate computational speed but also integrate the feature extraction process and forward propagation of the GCN into one computational framework. We compared our proposed method with DCN in our previous work [1]. Figure 16 shows the computational cost comparison of different methods—GCN1 denotes the process of the first step without GPU acceleration and GCN2 denotes the process of the first step with GPU acceleration.

As we can see, the duration of the feature extraction process was greatly reduced by using the redesigned calculation operation. Though the proposed method involves much more complex calculation, it can achieve a better performance while maintaining a computation cost close to that of the DCN.

## 4. Conclusions

In this paper, we presented a novel feature fusion method based on GCN for the AMC of underwater acoustic communication signals. The experimental results indicate that the proposed method can integrate multi-domain features and deep features to achieve a state-of-the-art AMC performance. The conclusions are highlighted as follows:

(1) To improve the stability and robustness of AMC in underwater scenarios, a new feature fusion method based on a graph convolution network was proposed to fuse the multi-domain features and deep features of underwater acoustic communication signals. The feature extraction methods and deep learning methods were effectively integrated into the constructed feature fusion framework.
(2) A graph was built for the multi-domain features and deep features based on their properties. The proposed feature fusion method can make full use of the relationships among these features. The experiments have shown that making use of the relationships can improve the AMC performance.
(3) The comparative experiments indicate that the feature fusion method based on GCN can significantly improve the AMC performance in underwater scenarios and achieve excellent classification performance in different simulated and real-world underwater acoustic channels.

## Figures and Tables

**Table 1:** Relationships among the HOC features.

	$C_{20}$	$C_{21}$	$C_{40}$	$C_{41}$	$C_{42}$	$C_{60}$	$C_{61}$	$C_{63}$	$C_{80}$
** $C_{20}$ **	-								
** $C_{21}$ **	∘	-							
** $C_{40}$ **	•	∘	-						
** $C_{41}$ **	•	•	∘	-					
** $C_{42}$ **	•	•	∘	∘	-				
** $C_{60}$ **	•	∘	•	∘	∘	-			
** $C_{61}$ **	•	•	•	•	∘	∘	-		
** $C_{63}$ **	•	•	∘	∘	•	∘	∘	-	
** $C_{80}$ **	•	∘	•	∘	∘	•	∘	∘	-

∘: has no relationship, •: has relationship, -: not available.

**Table 2:** The node–feature pairs.

Node	Feature	Node	Feature	Node	Feature
$v_{1}$	x(t)	$v_{6}$	$C_{41}$	$v_{11}$	$C_{80}$
$v_{2}$	F(x)	$v_{7}$	$C_{42}$	$v_{12}$	I(α)
$v_{3}$	$C_{20}$	$v_{8}$	$C_{60}$	$v_{13}$	I(f)
$v_{4}$	$C_{21}$	$v_{9}$	$C_{61}$	$v_{14}$	$U^{2}\left(f\right)$
$v_{5}$	$C_{40}$	$v_{10}$	$C_{63}$	$v_{15}$	$U^{4}\left(f\right)$

**Table 3:** Parameters of each modulation type.

Modulation Type	Sampling Rate (kHz)	Carrier Frequency Offset (Hz)	Symbol Rate (Baud)	Roll off Value	SNR (dB)
FSK	12k	300	100∼200	-	−9∼21
PSK	12k	300	800∼1200	0.1∼0.4	−9∼21
QAM	12k	300	800∼1200	0.1∼0.4	−9∼21

**Table 4:** Summary of the comparison of different feature sets.

	AMC Accuracy		
Feature Sets	Ch1	Ch2	Average
**All features**	**82.9%**	**81.4%**	**82.2%**
Without time domain	59.3%	50.8%	55.1%
Without STFT	79.7%	71.6%	75.7%
Without HOC	74.3%	73.5%	73.9%
Without CS	78.7%	78.9%	78.8%
Without HOM	79.2%	78.1%	78.7%

**Table 5:** Comparison of the influence of the edges inside HOC.

	AMC Accuracy		
Features Set	Ch1	Ch2	Average
**With HOC edges**	**82.9%**	**81.4%**	**82.3%**
Without HOC edges	79.5%	78.4%	79.0%

**Table 6:** Average accuracy of different methods in two channels.

Channel	Ch1	Ch2	Average	Channel	Ch1	Ch2	Average
GCN	**82.9**%	**81.4%**	**82.2%**	InceptionV3	74.5%	73.4%	74.0%
VGGnet	69.3%	76.0%	72.7%	GAN	78.8%	77.5%	78.2%
ResNet	68.5%	70.4%	69.5%	HOC	59.7%	69.6%	64.7%
LSTM	73.2%	69.2%	71.2%	CS	60.6%	63.6%	62.1%
DCN	74.9%	78.1%	76.5%	CWT	61.7%	65.3%	63.5%
CNN	68.4%	69.0%	69.7%				

**Table 7:** Classification results of the real-world underwater acoustic communication signals.

	2FSK	4FSK	BPSK	QPSK	16QAM	32QAM
Accuracy	84%	80%	77%	71%	67%	73%

## Data Availability

The data presented in this paper are available after contacting the corresponding author.

## Abbreviations

The following abbreviations are used in this manuscript:

AMC	Automatic modulation classification
GCN	Graph convolution network
HOC	High-order cumulant
CS	Cyclostationary statistics
DNN	Deep neural networks
CNN	Convolution neural network
GAN	Generative adversarial networks
RNN	Recurrent neural network
LSTM	Long short term memory
GRU	Gate recurrent unit
HOM	high order moment
SCD	Spectral correlation density
SCF	Spectral coherence function
STFT	Short-time Fourier transform
WGN	white Gaussian noise
FSK	Frequency shift keying
PSK	Phase shift keying
QAM	Quadrature amplitude modulation
DCN	Deep complex networks
